# Prediction of atherosclerotic cardiovascular disease mortality in a nationally representative cohort using a set of risk factors from pooled cohort risk equations

**DOI:** 10.1371/journal.pone.0175822

**Published:** 2017-04-20

**Authors:** Zefeng Zhang, Cathleen Gillespie, Barbara Bowman, Quanhe Yang

**Affiliations:** Division for Heart Disease and Stroke Prevention, National Center for Chronic Disease Prevention and Health Promotion, Centers for Disease Control and Prevention, Atlanta, GA, United States of America; University of Adelaide, AUSTRALIA

## Abstract

The American College of Cardiology/American Heart Association developed Pooled Cohort equations to estimate atherosclerotic cardiovascular disease (ASCVD) risk. It is unclear how well the equations predict ASCVD mortality in a nationally representative cohort. We used the National Health and Nutrition Examination Survey (NHANES) 1988–1994 and Linked Mortality through 2006 (n = 6,644). Among participants aged 40–79 years without ASCVD at baseline, we used Cox proportional hazard models to estimate the 10-year probability of ASCVD death by sex and race-ethnicity (non-Hispanic white (NHW), non-Hispanic black (NHB) and Mexican American (MA)). We estimated the discrimination and calibration for each sex-race-ethnicity model. We documented 288 ASCVD deaths during 62,335 person years. The Pooled Cohort equations demonstrated moderate to good discrimination for ASCVD mortality, with modified C-statistics of 0.716 (95% CI 0.663–0.770), 0.794 (0.734–0.854), and 0.733 (0.654–0.811) for NHW, NHB and MA men, respectively. The corresponding C-statistics for women were 0.781 (0.718–0.844), 0.702 (0.633–0.771), and 0.789 (CI 0.721–0.857). Modified Hosmer-Lemeshow χ^2^ suggested adequate calibration for NHW, NHB and MA men, and MA women (p-values: 0.128, 0.295, 0.104 and 0.163 respectively). The calibration was inadequate for NHW and NHB women (p<0.05). In this nationally representative cohort, the Pooled Cohort equations performed adequately to predict 10-year ASCVD mortality for NHW and NHB men, and MA population, but not for NHW and NHB women.

## Introduction

The American College of Cardiology (ACC) and American Heart Association (AHA) released new guidelines on the assessment of cardiovascular risk in 2013 [1]. In this guideline, new equations for the 10-year risk of developing atherosclerotic cardiovascular disease (ASCVD), the Pooled Cohort risk equations [1], were developed. These new risk equations were derived from five National Heart, Lung, and Blood Institute-funded, racially and geographically diverse prospective cohort studies. Ten-year risk was defined as the risk of developing a first hard ASCVD event, including first occurrence of nonfatal myocardial infarction, nonfatal stroke, fatal coronary heart disease (CHD), or fatal stroke among people aged 40 to 79 years without ASCVD at baseline.

Since their release, some studies suggested that the ACC-AHA Pooled Cohort risk equations tended to overestimate risk in other independent cohorts. Further, the overestimation could not be completely explained by enhanced identification of study outcomes, increased use of statins, and arterial revascularization procedures [2–4]. It is unknown how well the Pooled Cohort equations risk factors and their interactions might predict ASCVD mortality in a nationally representative cohort. We used the National Health and Nutrition Examination Survey (NHANES) 1988–1994 (III) and NHANES Linked Mortality File through 2006, and applied the set of risk factors from the Pooled Cohort risk equations to predict ASCVD mortality in this nationally representative cohort. We evaluated calibration and discrimination of the risk prediction equations by sex and race-ethnicity groups.

## Methods

NHANES uses a complex, stratified, multistage probability cluster sampling, cross-sectional design to collect health and nutritional data from a representative sample of the noninstitutionalized US population. The design and operation of NHANES have been described elsewhere [5]. We used data from NHANES III (1988–1994). Of 8,495 participants who were 40–79 years of age and attended the Mobile Examination Center, we sequentially excluded 5 pregnant women; 4 participants who were not eligible for follow-up; 133 participants who had body mass index (BMI) less than 18.5 (calculated as weight in kilograms divided by height in meters squared); 974 participants who reported a history of heart attack (n = 565), stroke (n = 234), or congestive heart failure (n = 175); 337 participants who had cancer at baseline, and 397 participants with missing values for the variables used in the Pooled Cohort risk equations, leaving 6,644 adults for analysis. Study protocols for NHANES were approved by the National Center for Health Statistics ethics review board. Signed informed consent was obtained from all participants.

We used NHANES III (1988–1994) Linked Mortality Files that were linked through 2006 with a probabilistic matching algorithm to the National Death Index to determine mortality status. Follow-up of participants continued until death attributable to ASCVD at or less than 10 years, with censoring at the time of death for those who died from causes other than ASCVD, or for those who survived after 10 years of follow-up. Participants not matched with a death record were considered to be alive through the entire follow-up period. A detailed description of this methodology has been published elsewhere [6]. The International Statistical Classification of Diseases, 10th Revision was used to identify participants for whom ASCVD (codes I58-I63, I70 and I71) was listed as the underlying cause of death.

Race-ethnicity was categorized into non-Hispanic white (NHW), non-Hispanic black (NHB), Mexican-American, or other. Participants were classified as current smokers if they reported ever smoking ≥ 100 cigarettes during the entire life and that they currently smoked every day or some days, or if their serum cotinine was > 10 ng/mL. Participants were classified as having diabetes if they answered yes to the question, “Have you ever been told by a doctor that you have diabetes or sugar diabetes?”, or had a fasting glucose ≥126 mg/dL, or HbA1c ≥6.5%. Other risk factors include total cholesterol, high-density lipoprotein cholesterol (HDL-C), systolic blood pressure, and anti-hypertensive medication use. Participants were classified as having hypertension if they had a mean systolic blood pressure ≥140 mmHg, a mean diastolic blood pressure ≥90 mmHg, or if they reported currently taking antihypertensive medications. Mean blood pressure was calculated as the average of up to 3 readings obtained under standard conditions during a single physical examination [7]. The standardized laboratory procedures to measure lipid levels have been described elsewhere [8].

We evaluated the discrimination and calibration of predicting 10-year ASCVD mortality using the same set of risk factors and interaction terms from the Pooled Cohort risk equations. Discrimination is measured by calculating the area under the receiver operating characteristics curve statistic or C-statistic, and assesses the ability of the risk score to differentiate between participants who died from ASCVD at 10-year follow up and those who didn’t [9,10]. Calibration assesses the agreement between the predicted and observed 10-year ASCVD mortality, by plotting predicted probabilities versus the observed proportions of ASCVD deaths at 10-year follow-up [11]. We also calculated R^2^, which measures the explained variation in the survival analysis [12].

Data on characteristics were expressed as means and standard errors for continuous variables or as percentages and standard errors for categorical variables and were compared by sex. A *t-*test was used for comparisons between sex groups for continuous variables. The *χ*^2^ test was used for categorical variables. We used Cox proportional hazard models to estimate gender and race-ethnicity specific β-coefficients (i.e., NHW, NHB, and Mexican-American men, and women respectively) for the risk factors used in the Pooled Cohort risk equations, which included age, current smoking, diabetes, systolic blood pressure, use of antihypertensive medication, total cholesterol, and HDL-C. We then calculated the 10-year predicted risk of ASCVD mortality by sex and race-ethnicity using these coefficients. We calculated observed and predicted ASCVD mortality rate at 10 years within quartiles or tertiles of predicted risk, depending on the the sample size of the sex and race-ethnicity groups. Overall, 95.7% of the NHANES participants were censored at 10 years of follow-up free of ASCVD deaths.

We calculated C-statistics and R^2^ to assess discrimination of the Pooled Cohort equations risk factors. A C-statistic between 0.70 and 0.80 is considered as moderate to good and 0.80 or greater is considered as excellent discrimination [13]. The calibration was determined by comparing the observed and predicted number of 10-year ASCVD deaths. We tested the calibration significance using the modified Hosmer-Lemeshow statistic [11]. A p-value of less than 0.05 indicates poor calibration.

We performed sex and race/ethnicity-specific analyses (for NHW, NHB and Mexican-American), and applied the set of risk factors and their interactions for NHW to Mexican American population [1]. We used SUDAAN 11 (RTI International, Research Triangle Park, NC) for all analyses to account for the complex sampling design. We used Stata 13 to calculate R^2^. All tests were two-sided, and a *p-*value of <0.05 was considered statistically significant.

## Results

The average follow-up period for the analytic sample was 9.38±1.83 years. The average age of the participants at baseline was 54.7 years, and over 80% were NHW. Around 29.3% of participants were current smokers, 11.3% had diabetes, about 26% had hypertension, and 18.2% of participants were taking antihypertensive medications. Mean treated and untreated systolic blood pressure were 125.2 mmHg and 139.9 mmHg, respectively. The mean total cholesterol and HDL-C were 217.0 mg/dL and 50.9 mg/dL (45.4 mg/dL in men and 55.9 mg/dL in women), respectively. Compared to women, men were younger and were more likely to be white, to be current smoker, to have hypertension, and to have lower levels of total cholesterol and HDL-C (Table 1).

**Table 1:** Characteristics of adults aged 40–79 years with no prior atherosclerotic cardiovascular disease, NHANES III linked mortality file 1988–2006.

	Male (n = 3176)	Female (n = 3468)	P value
**Age, mean (years, se)**	54.0 (0.32)	55.3 (0.38)	<0.001
**Race/ethnicity**			
Non-Hispanic white (%, se)	81.0 (1.28)	79.7 (1.40)	0.035
Non-Hispanic black (%, se)	8.6 (0.48)	9.7 (0.64)	
Mexican-American (%, se)	4.0 (0.38)	3.6 (0.26)	
Other (%, se)	6.5 (1.08)	7.0 (1.08)	
Current smoker* **(%, se)**	36.8 (1.26)	22.5 (1.12)	<0.001
Diabetes* **(%, se)**	12.2 (0.73)	10.5 (0.98)	0.127
Hypertension* **(%, se)**	27.6 (1.27)	24.5 (1.23)	0.045
**Total cholesterol** (mg/dL)	212.4 (1.25)	221.1 (1.17)	<0.001
**High-density lipoprotein** (mg/dL)	45.4 (0.49)	55.9 (0.51)	<0.001

* Current smokers were defined if the participants reported ever smoking ≥ 100 cigarettes during the entire life and that they currently smoked every day or some days, or if their serum cotinine was > 10 ng/mL. Participants were classified as having diabetes if they answered yes to the question “Have you ever been told by a doctor that you have diabetes or sugar diabetes?” or have fasting glucose ≥126 mg/dL or A1c ≥6.5%. Participants were classified as having hypertension if they had a mean systolic blood pressure ≥140 mmHg, a mean diastolic blood pressure ≥90 mmHg, or taking antihypertensive medications.

**Fig 1:**
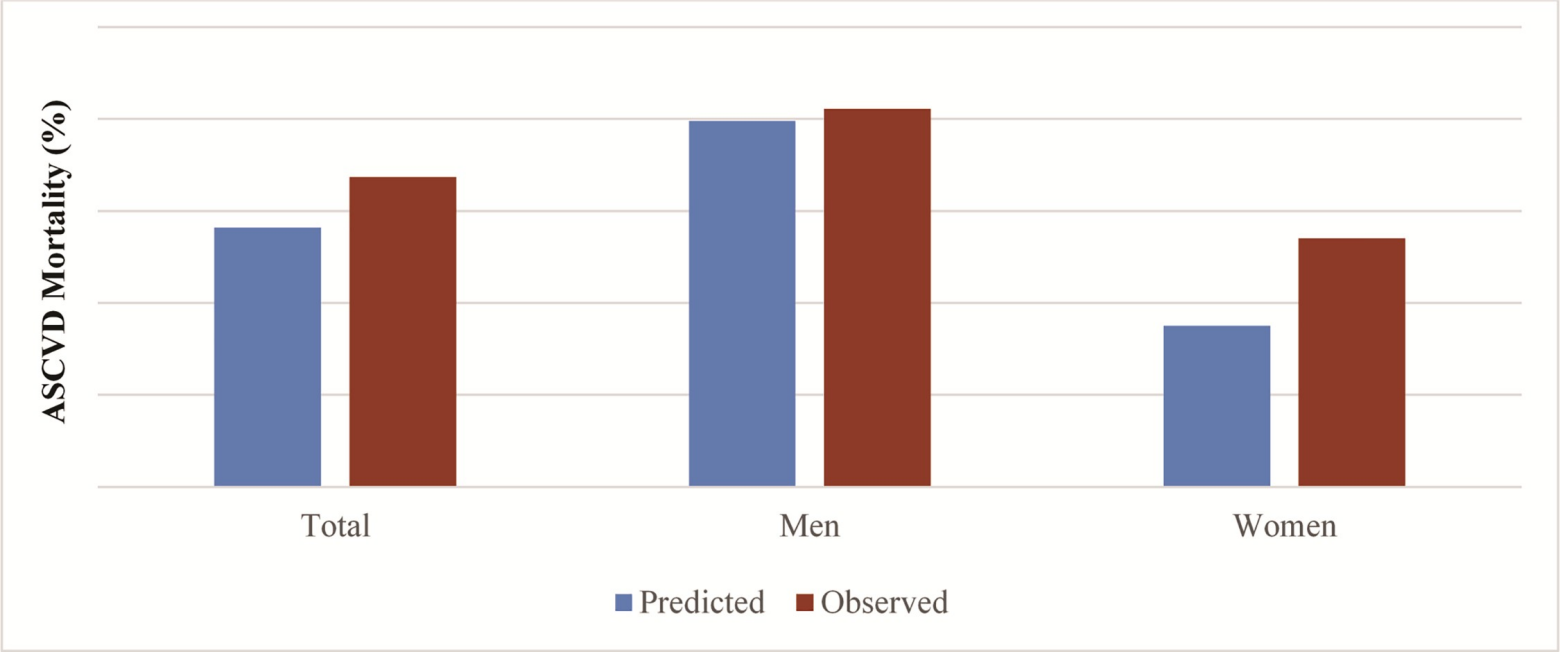
The observed and predicted 10-year ASCVD mortality overall and by sex.

Table 2 shows the coefficients for the risk factors from the Pooled Cohort equations. Throughout the 10-year follow-up, we documented 288 ASCVD deaths (156 men and 132 women) during 62,335 person years. The observed (based on Kaplan-Meier curve) and predicted 10-year ASCVD death rates were 3.37% (95% CI, 2.75%-4.13%) and 2.82%, respectively (Fig 1). The observed and predicted 10-year ASCVD death rates were 4.11% (95% CI, 3.13%-5.40%) and 3.98% in men, and 2.70% (95% CI, 2.07%-3.53%) and 1.75% in women, respectively.

**Table 2:** Sex and race/ethnicity-specific equation parameters for estimation of 10-year risk of ASCVD mortality, NHANES III linked mortality file 1988–2006.

	NHW		NHB		Mexican American	
	β-coefficient	P value	β-coefficient	P value	β-coefficient	P value
**Male**	(n = 1412)		(n = 793)		(n = 858)	
Ln Age (y)	29.05	0.1637	5.50	0.0007	19.52	0.2050
Ln Age squared						
Ln total cholesterol (mg/dL)	27.75	0.0524	0.12	0.8739	19.16	0.2431
Ln age*Ln total cholesterol	-6.70	0.0510			-4.92	0.2409
Ln HDL-c (mg/dL)	-15.58	0.1018	1.49	0.0242	-12.60	0.4059
Ln Age*Ln HDL-c	3.78	0.0975			2.87	0.4678
Ln treated systolic BP (mmHg)	3.61	0.0052	1.16	0.3836	3.28	0.2123
Ln Age*Ln treated systolic BP						
Ln untreated systolic BP (mmHg)	3.60	0.0057	1.25	0.3585	3.36	0.2147
Ln Age*Ln untreated systolic BP						
Current smoker (1 = Yes, 0 = No)	9.85	0.2424	1.08	0.0023	4.00	0.7099
Ln Age*current smoker	-2.25	0.2608			-0.82	0.7568
Diabetes (1 = Yes, 0 = No)	0.32	0.2317	0.55	0.1201	0.48	0.3047
**Female**	(n = 1585)		(n = 896)		(n = 827)	
Ln Age (y)	48.06	0.5456	86.56	0.2549	88.97	0.0047
Ln Age squared	-5.72	0.6065			0.68*	0.4729
Ln total cholesterol (mg/dL)	4.38	0.8422	-1.37	0.0558	40.12	0.1168
Ln age*Ln total cholesterol	-0.86	0.8666			-9.02	0.1337
Ln HDL-c (mg/dL)	-21.98	0.1778	5.19	0.8359	43.38	0.0295
Ln Age*Ln HDL-c	5.13	0.1783	-1.04	0.8631	-10.30	0.0285
Ln treated systolic BP (mmHg)	1.73	0.2131	65.10	0.1706	2.56	0.3348
Ln Age*Ln treated systolic BP			-15.35	0.1715		
Ln untreated systolic BP (mmHg)	1.64	0.2391	65.38	0.1816	2.47	0.3542
Ln Age*Ln untreated systolic BP			-15.42	0.1819		
Current smoker (1 = Yes, 0 = No)	50.21	0.0374	0.41	0.2046	-26.17	0.0092
Ln Age*current smoker	-11.49	0.0428			6.32	0.0086
Diabetes (1 = Yes, 0 = No)	0.61	0.1546	0.82	0.0318	0.68	0.2644

BP = blood pressure; HDL-c = High-density lipoprotein cholesterol; Ln = natural log

* the interger of natural log age square is used.

Table 3 summarizes the discriminative ability of each model. The C-statistics were 0.716 (95% CI, 0.663–0.770), 0.794 (95% CI, 0.734–0.854), and 0.733 (95% CI, 0.654–0.811), respectively, for NHW, NHB and Mexican American men. The corresponding C-statistics were 0.781 (95% CI, 0.718–0.844), 0.702 (95% CI, 0.633–0.771), and 0.789 (95% CI, 0.721–0.857) for NHW, NHB and MA women, respectively. The R^2^ ranged from 0.176 to 0.276 for NHW, NHB and Mexican American men, and 0.202 to 0.386 for NHW, NHB and Mexican American women.

**Table 3:** Measurements of discrimination by sex and race/ethnicity, NHANES III linked mortality file 1988–2006.

	C-Statistic	R^2^
**Male**		
Non-Hispanic white	0.716 (0.663–0.770)	0.176 (0.009–0.433)
Non-Hispanic black	0.794 (0.734–0.854)	0.276 (0.151–0.583)
Mexican American	0.733 (0.654–0.811)	0.256 (0.149–0.664)
**Female**		
Non-Hispanic white	0.781 (0.718–0.844)	0.386 (0.253–0.661)
Non-Hispanic black	0.702 (0.633–0.771)	0.202 (0.126–0.545)
Mexican American	0.789 (0.721–0.857)	0.301 (0.154–0.738)

**Fig 2:**
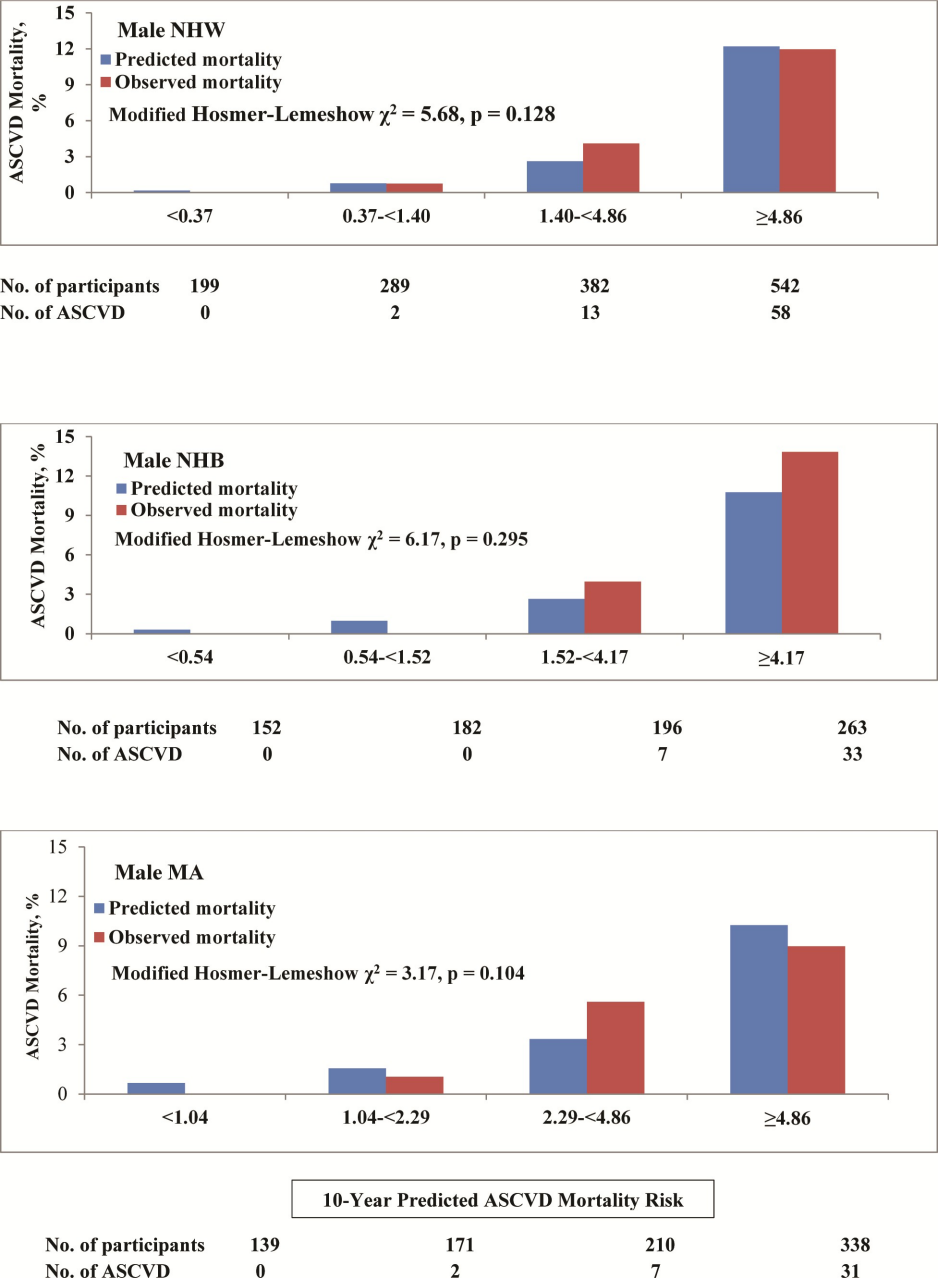
Calibration of prediction models by race-ethnicity in men, NHANES III linked mortality file, 1988–2006.

**Fig 3:**
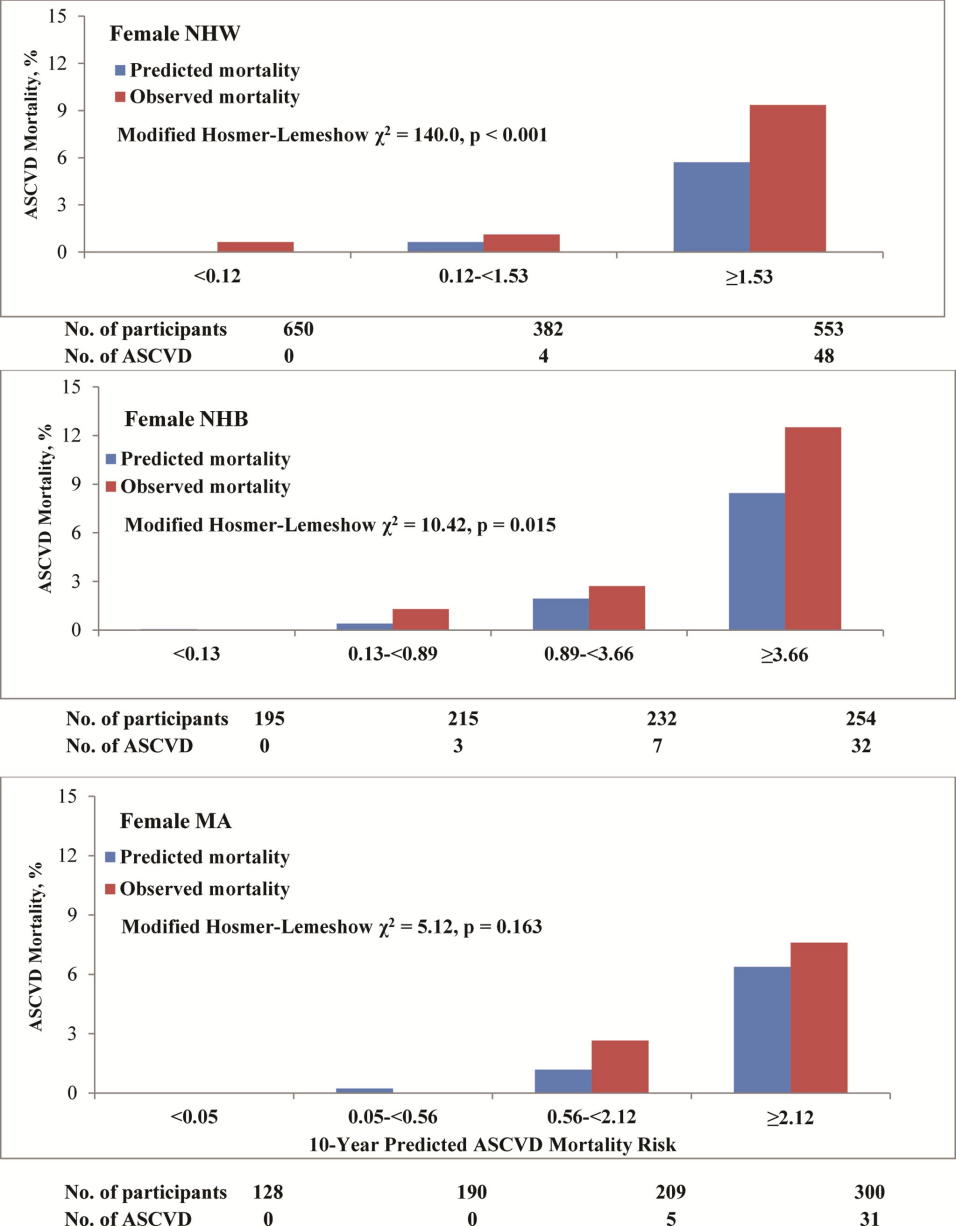
Calibration of prediction models by race-ethnicity in women, NHANES III linked mortality file, 1988–2006.

Figs 2 and 3 compared the average 10-year predicted ASCVD mortality with the observed 10-year ASCVD mortality by sex and race-ethnicity groups and quartile or tertile of predicted risk. The calibration χ^2^ statistics were 5.68, 6.17, and 3.17 with p-values of 0.128, 0.295 and 0.104 for NHW, NHB and Mexican American men, respectively, indicating statistically acceptable calibration. The calibration χ^2^ statistics were 140.0, 10.42, and 5.12 with p-values of <0.001, 0.015 and 0.163 for NHW, NHB and Mexican American women, respectively, indicating statistically acceptable calibration in Mexican American women and inadequate calibration for NHW and NHB women. The Pooled Cohort risk equations tended to underestimate ASCVD mortality in both NHW and NHB women, particularly in the highest 10-year predicted mortality risk group (Fig 3).

## Discussion

In this large, nationally representative sample of the U.S. men and women aged 40–79 years without cardiovascular disease at baseline, we estimated the coefficients for the set of risk factors used in the Pooled Cohort risk equations, and calculated the predicted 10-year ASCVD mortality. Our findings suggested that the set of risk factors and their interactions used in the Pooled Cohort risk equations had reasonable discrimination ability for 10-year ASCVD mortality by sex and race/ethnicity, and the calibration of the predicted models was adequate for NHW, NHB and Mexican American men and Mexican American women, but inadequate for NHW and NHB women.

Compared with the National Cholesterol Education Program, Adult Treatment Panel III (ATP III) guidelines which only included 10-year risk of CHD, the Pooled Cohort risk equations from the 2013 ACC-AHA guideline expand the endpoint to include risk of all hard ASCVD, including CHD and stroke. It also includes diabetes as a predictor. In addition, the 2013 guideline lowers risk threshold for statin treatment from 20% CHD risk in the ATP-III [14] guidelines to 7.5% ASCVD risk. Several studies assessed the accuracy of the Pooled Cohort risk equations in predicting 10-year risk of developing ASCVD [15–20]. Ridker and Cook [3] suggested that the new Pooled Cohort risk equations systematically overestimated observed risk by 75–150%, roughly doubling the actual risk in three large-scale primary prevention cohorts: the Women’s Health Study, the Physicians’ Health Study, and the Women’s Health Initiative Observational Study. Kavousi et al [4] used a cohort of Dutch individuals aged 55 years or older and reported that based on Pooled Cohort risk equations, the 2013 ACC-AHA guidelines on the treatment of blood cholesterol to reduce ASCVD [21] would recommend statin treatment for nearly all men and two-third of women, exceeding the proportions based on the recommendations by ATP-III or European Society of Cardiology guidelines. Because the Pooled Cohort risk prediction score serves as a guide to inform clinical decisions for statin therapy, these studies have raised the concern that the overestimation of predicted risk might result in overprescription of statins, and could lead to more than a billion people aged 40–79 years taking statins worldwide [15].

Our analyses, using a nationally representive cohort, showed that the risk factors used in the Pooled Cohort risk equations had reasonable discrimnination ability for ASCVD mortality for all sex and race-ethnicity-specific prediction models. The C-statistic ranged from 0.702 to 0.794. Our findings were consistent with the results from the Reason for Geographic and Racial Differences in Stroke cohort study that reported C-statistics 0.75, 0.66, 0.69 and 0.74 for women, men, blacks and whites, respectively for 10-year risk of developing ASCVD [22]. However, Kavousi et al [4] reported the C-statistic of 0.67 and 0.68 in men and women for developing ASCVD when the Pooled Cohort risk equations were assessed in Dutch population aged 55 to 75 years [4]. Accurate calibration of the risk prediction model is important because individual predicted risk is usually used in clinical decision making for treatment initiation. Our calibration analyses showed reasonably good agreement between predicted and actual ASCVD mortality rates for NHW and NHB men, and Mexcican American men and women, but not for NHW and NHB women. In NHW and NHB women, the Pooled Cohort equations prediction functions underestimated ASCVD mortality, particularly in the high predicted-risk group. The reason for inadequate calibration in NHW and NHB women is unclear, the small number of women with events might play a role.

Our results might not be directly comparable to the results of other cohort studies using the Pooled Cohort Risk Equations. First, the population of our cohort differed from the cohorts used to derive or validate the Pooled Cohort Risk Equations. For example, the Women’s Health Study and Women’s Health Initiative Observational Study included women only, and the Physicians’ Health Study only included physicians, whereas our cohort included both men and women, and different race-ethnicity groups. Second, unlike other studies that applied the Pooled Cohort Risk Equations to their cohorts, we estimated the β-coefficients for each risk factor and the interactions between the risk factors used in Pooled Cohort Risk Equations, then calculated the predicted ASCVD mortality using these coefficients. Our main objective was to evaluate the set of risk factors used in the Pooled Cohort Risk Equations in predicting the ASCVD death in a nationally representative sample by sex and race/ethnicity groups. Third, our outcome included only fatal CHD and stroke, not nonfatal myocardial infarction, or stroke.

To our knowledge, this is the first study to examine how well the set of the risk factors and their interactions from the Pooled Cohort risk equations predict ASCVD mortality using a nationally representative cohort of US adults. Our study included all risk factors needed for each of the prediction models, and all risk factors were collected or measured using standardized methods. NHANES III Linked Mortality File also had accurate long term follow-up of mortality status. Using this nationally representative cohort, our findings could be generalizabilzed to U.S. non-Hispanic whites, non-Hispanic blacks and Mexican Americans.

Our study has several limitations. First, the Pooled Cohort risk equations predict the 10-year risk of developing both fatal and non-fatal ASCVD; our study focused only on fatal ASCVD as non-fatal ASCVD events are not captured in NHANES. Second, prevalent diseases, such as a history of heart attack, stroke, and congestive heart failure are self-reported. Also, the demographic composition of the U.S. population has changed over the past 2 decades. Approximately 80% of our analyzed cohort (NHANES 1988–1994) consisted of non-Hispanic whites, while in NHANES 2009–2014, only 71% of the population was non-Hispanic whites based on the same exclusion criterion. Thus, our results might not be representative of the contemporary U.S. population. Lastly, our study captured a limited number of ASCVD free participants and a limited number of events for in certain sex and racial/ethnic strata for risk predictions, and this limitation might partly explain the inadequate calibaration among NHW and NHB women.

The Pooled Cohort risk equations were designed to predict the 10-year risk of developing ASCVD in the population aged 40–79 years. Our findings suggest that the same set of risk factors and their interactions from the Pooled Cohort equations perform adequately to predict 10-year risk of ASCVD mortality among adult NHW and NHB men, and MA population in the United States, but not for NHW and NHB women.

## Supporting information

S1 Data SAS dataset used in the analyses. (SAS7BDAT)
